# Evaluation of Factors Affecting Colostrum Quality and Quantity in *Holstein* Dairy Cattle

**DOI:** 10.3390/ani11072005

**Published:** 2021-07-05

**Authors:** Aikaterini Soufleri, Georgios Banos, Nikolaos Panousis, Dimitrios Fletouris, Georgios Arsenos, Alexandros Kougioumtzis, Georgios E. Valergakis

**Affiliations:** 1 Laboratory of Animal Husbandry, Faculty of Veterinary Medicine, School of Health Sciences, Aristotle University of Thessaloniki, Box 393, 54124 Thessaloniki, Greece; banos@vet.auth.gr (G.B.); arsenosg@vet.auth.gr (G.A.); akougiou@vet.auth.gr (A.K.); geval@vet.auth.gr (G.E.V.); 2 Scotland’s Rural College, Roslin Institute, Building, Easter Bush, Edinburgh EH25 9RG, UK; 3 Clinic of Farm Animals, Faculty of Veterinary Medicine, School of Health Sciences, Aristotle University of Thessaloniki, 54124 Thessaloniki, Greece; panousis@vet.auth.gr; 4 Laboratory of Safety and Quality of Dairy Foods, Faculty of Veterinary Medicine, School of Health Sciences, Aristotle University of Thessaloniki, 54124 Thessaloniki, Greece; djflet@vet.auth.gr

**Keywords:** dairy cattle, colostrum composition, factors, refractometer

## Abstract

**Simple Summary:**

Administration of high-quality colostrum is vital for short and long-term health of newborn calves. It ensures the passive transfer of immunity and covers the high energy requirements during the first hours of calf’s life. Colostrum composition and factors affecting it have not been evaluated in large scale studies. Several factors affecting colostrum quality and yield were revealed. The digital BRIX refractometer proved quite useful. Colostrum quality can be maximized by management practices accounting for factors affecting its composition.

**Abstract:**

The objective of this study was to conduct a large-scale investigation of colostrum composition and yield and an evaluation of factors affecting them. In this study, 1017 clinically healthy Holstein cows from 10 farms were used. The colostrum TS were measured using a digital Brix refractometer. Fat, protein and lactose content were determined using an infrared Milk Analyzer. Statistical analysis was conducted using a series of univariate general linear models. The mean (±SD) percentage of colostrum fat, protein, lactose and TS content were 6.37 (3.33), 17.83 (3.97), 2.15 (0.73) and 25.80 (4.68), respectively. Parity had a significant positive effect on the protein and TS content and a negative one on fat content. The time interval between calving and colostrum collection had a significant negative effect on the fat, protein and TS contents and a positive one on lactose. Colostrum yield had a significant negative effect on the protein and TS content, and it was affected by all factors considered. In addition to TS, the evaluation of the colostrum fat content appears essential when neonates’ energy needs are considered. The Brix refractometer, an inexpensive and easy to use devise, can be used effectively in colostrum quality monitoring.

## 1. Introduction

The administration of adequate quantities of high-quality colostrum to calves as soon as possible after parturition is a very important management practice that assures their survival, health and future production [1,2,3]. Colostrum contains nutrients, growth factors, cytokines and immunoreactive cells [4,5]; its composition varies markedly among cows and farms [6,7].

Most researchers have focused on the immunological quality of colostrum and the factors affecting it [8,9,10], as it ensures the passive transfer of immunity and calf protection from a wide range of infectious diseases, until they are capable of producing their own antibodies at 3 to 6 weeks of age [11,12]. Immunoglobulin G (IgG) content is affected by several factors including breed [13,14], age and parity of the dam [9,15], length of the dry period [15], vaccination schemes [16], colostrum yield [17] and the time between calving and colostrum collection [8,9].

Radial immunodiffusion (RID) is the golden standard method for the analysis of colostrum IgG content, but it is expensive and time consuming [18,19]. The assessment of colostrum IgG content by measuring total solids (TS) concentration using a Brix refractometer is an indirect but reliable, inexpensive, and easy to use cowside method [20,21].

Colostrum composition is also essential for thermogenesis and maintenance of body temperature because neonates are born with low energy reserves [22,23]. To date, few studies have focused on colostrum composition mostly based on a limited sample size [4,24,25]. Large scale epidemiological studies that have as their main purpose the investigation of full colostrum composition and factors affecting it are scarce in the literature [7,26].

The objective of the present study was to perform a large-scale investigation of colostrum composition of Holstein cows and evaluate factors that affect yield and the content of all major colostrum components.

## 2. Materials and Methods

The research was conducted in compliance with institutional guidelines and approved by the Research Committee of the Aristotle University of Thessaloniki, Thessaloniki, Greece. All farmers gave informed consent for the cows to be included in the study and the testing procedures.

### 2.1. Animals and Management

The study was conducted in 10 commercial dairy herds in Northern Greece; farms were visited from February 2015 to September 2016 for sample collection and data recording. Herd size ranged from 90 to 300 dairy cows and a total of 1074 healthy Holstein cows were sampled once during the test period. Herds followed a year-round calving pattern and had a high level of production (8000–10,000 kg of milk per cow per year). Parity distribution was 387, 311, 181 and 195 cows for parities 1, 2, 3 and ≥4, respectively. Dry cows were housed in straw yards, as a single group on six farms and in two separate groups (far off and close up) on four farms. All cows were fed total mixed rations that included corn silage, wheat straw, soybean meal and a mineral/vitamin supplement formulated to meet or exceed net energy lactation (NEL) and metabolizable protein (MP) requirements according to NRC (2001) recommendations.

### 2.2. Clinical Examination

All cows were clinically examined at the day of calving; those exhibiting any sign of disease or altered colostrum appearance (watery, clots, blood, etc.) were excluded from the study. At the same time, cows were body condition scored using the 1- to 5-point scale of Ferguson et al. [27] in increments of 0.25. In this scale, 1 corresponds to emaciated and 5 to obese animals.

### 2.3. Colostrum Sample Collection

All four udder quarters of healthy cows, which were not milked or suckled before, were completely milked by a portable milking machine into a steel churn. Time interval between calving and milking was recorded; depending on calving time, colostrum was collected from 10 to 960 min after calving. Colostrum was transferred into plastic graded milk buckets and weighted; the volume and weight were recorded (thereafter referred as colostrum yield). Colostrum from each cow was agitated for at least 2 min to ensure an even distribution of constituents; a 100 mL sample was collected and divided into 2 plastic vials (50 mL each). One was used for cowside analysis and the second, which was destined for chemical analysis, was properly labeled (farm identification number, dam ear tag and date of calving), placed in a cooler, transported to the Laboratory of Safety and Quality of Dairy Foods, School of Veterinary Medicine, Aristotle University of Thessaloniki, and frozen immediately at −20 °C.

### 2.4. Cowside Determination of Colostrum Quality

Colostrum TS were measured immediately after sampling using a digital Brix refractometer (PAL-1, ATAGO, Fukaya-shi, Saitama, Japan). This is an indirect assessment of the colostrum’s immunoglobulin concentration. The instrument measures the refractometric index of liquids on a Brix scale. It has a measurement range from 0–53%, is independent of ambient temperature and has high accuracy (±0.1%). Calibration of the instrument was carried out before every measurement, with distilled water. The vial containing the colostrum was agitated at least 10 times to ensure an even distribution of constituents and a quantity of 0.3 mL was placed on the prism surface. All measurements were performed in duplicate, recorded and then averaged. A measurement of ≥22% was considered indicative of high colostrum quality [20].

### 2.5. Laboratory Determination of Colostrum Composition

Frozen colostrum samples were thawed in a water bath at 40 ± 2 °C; vials were inverted 10 times to thoroughly mix the colostrum and secure an even distribution of constituents. Colostrum fat, protein and lactose content was determined using an infrared Milk Analyzer (MilkoScan™ Minor, Foss, Denmark) after a dilution 1:4 with distilled water. All analyses were performed at the Laboratory of Safety and Quality of Dairy Foods, School of Veterinary Medicine, Aristotle University of Thessaloniki.

### 2.6. Calculation of Colostrum Net Energy Content

Net energy content of colostrum was calculated according to the NRC (2001) [28] equation:

(1) $$(0.057\;\times \;\mathrm{Crude}\;\mathrm{protein}\left(\%\right)+0.092\;\times \;\mathrm{Fat}\left(\%\right)+0.0395\;\times \;\mathrm{Lactose}\left(\%\right){)}^{1}\;\times \;0.97^{2}\;\times \;0.96^{3}\;\times \;0.86^{4}$$

where:

^1^ Gross Energy

^2^ Digestible Energy = Gross Energy×0.97

^3^ Metabolizable Energy = Digestible Energy×0.96

^4^ Net Energy = Metabolizable Energy×0.86

### 2.7. Data Set

Cowside colostrum measurements, laboratory analysis results and routinely kept farm records were combined to build the project’s database. This included dry period length (for cows with parity ≥2), milk yield of previous 305-d lactation (for cows with parity ≥2), dry cow grouping (one or two groups), age at calving (in months), parity number, season of calving (spring, autumn, winter, summer), Body Condition Score (BCS) at calving, time interval between calving and collection of colostrum, colostrum yield, TS (Brix score) and fat, protein, lactose and energy content. The data set is described in Table 1.

### 2.8. Statistical Analysis

Colostrum TS, fat, protein, lactose and energy content and colostrum yield were statistically analyzed using univariate general linear models. These models included the farm as a random effect and calendar season of calving, parity number, age at calving, 305-d milk yield in previous lactation, dry period length, BCS, colostrum yield (for composition traits) and time interval between calving and colostrum collection as fixed effects. Dry period length was classified as follows: ≤45, 46–64, 65–84 and ≥85 days. Milk yield in previous 305-d lactation was classified as ≤7000, 7001–9000, 9001–11,000 and ≥11,001. Parity number was grouped as 1, 2, 3 and ≥4. Calendar season of calving was categorized as winter (December, January, February), spring (March, April, May), summer (June, July, August) and autumn (September, October, November). Colostrum yield was classified as ≤4, 4.1–8.4 and ≥8.5 kg. Time interval between calving and colostrum collection was grouped as ≤2, 2–6 and ≥6 h. Age at calving (recorded in months) and BCS were included in the analyses as covariates.

Each trait was analyzed separately using the following model:

(2) $$Y_{ijkmq}=HYS_{i}+L_{j}+{Μ}_{k}+\beta_{1}\times dur+\beta_{2}\times time+Q_{m}+\beta_{3}\times BCS+\beta_{4}\times milk+\beta_{5}\times age+e_{ijkmq}$$

where:

$Y_{ijkmq}$ is the dependent variable (colostrum trait record)

$HYS_{i}$ is the fixed effect of herd-year-season of calving *i* (80 levels)

$L_{j}$ the fixed effect of number of lactation *j* (4 levels)

${Μ}_{k}$ the fixed effect of calendar month when the record was taken *k* (12 levels)

$\beta_{1}$ linear regression on duration of previous dry period (dur)

$\beta_{2}$ linear regression on time colostrum collection (time)

*Q_m_* the fixed effect of colostrum quantity class m (3 levels; content traits only)

$\beta_{3}$ linear regression on BCS

$\beta_{4}$ linear regression on milk yield of previous 305-d lactation (milk)

$\beta_{5}$ linear regression on cow age at calving (age)

$e_{ijkmq}$ is the random residual term.

Statistical analyses were performed using the IBM SPSS software (IBM Corp. Released 2015, IBM SPSS Statistics for Windows, Version 23.0. IBM Corp, Armonk, NY, USA). In all cases, significance level was set at *p* ≤ 0.05.

## 3. Results

**Figure 1:**
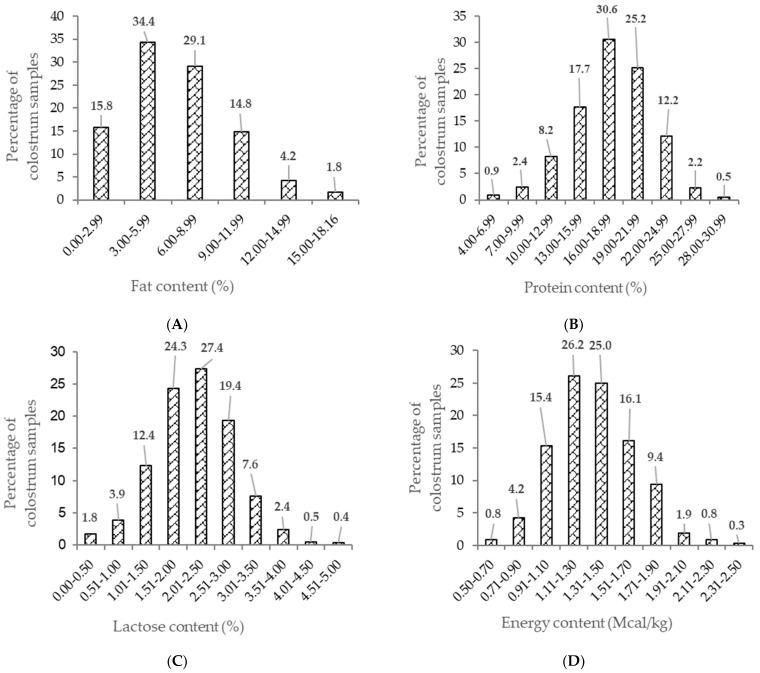
Distribution of fat (**A**), protein (**B**), lactose (**C**) and energy content (**D**) in colostrum samples.

**Figure 2:**
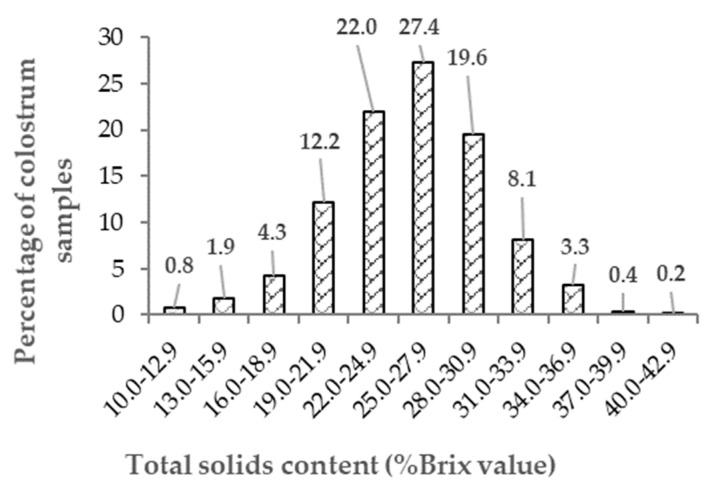
Distribution of total solids content (%Brix value) in 1074 colostrum samples.

The descriptive statistics of colostrum the fat, protein, lactose, TS and energy content are presented in Table 2. The mean % (±SD) concentrations were 6.37 (3.33)%, 17.83 (3.97)%, 2.15 (0.73)%, 25.80 (4.68)% and 1.35 (0.29) Mcal/kg, respectively. The distribution of the fat, protein, lactose and energy contents in the colostrum samples are shown in Figure 1 and those of TS (indirect estimate of immunoglobulin content) in Figure 2; all were normally distributed (skewness and kurtosis). The colostrum fat content had a high coefficient of variation (52%). The coefficient of variation of the lactose content was 34%, and those of the protein and energy contents were around 22%. The TS concentration ranged from 10.70% to 41.40%, and 19.2% of samples were classified as being of poor quality (TS < 22%). The colostrum yield ranged from 0.5 kg to 23.5 kg with a mean of 6.2 kg and the coefficient of variation (61%) was high.

The descriptive statistics of the colostrum components and energy content, among and within farms, are presented in Table 3. Among farms, the variability for the fat, protein, lactose, TS and energy content was low and ranged from 6% to 22%. However, within farms, variability was rather high for fat and lactose content, ranging from 44% to 63% and from 28% to 82%, respectively. For the protein, TS and energy contents, variability within farms was low and ranged from 6% to 16%. Regarding the colostrum yield, the among farm variability was 35%; the within farm variability ranged from 25% to 74%.

### Effects on Colostrum Quality and Yield

Effects of the factors included in the model on the colostrum fat, protein, lactose, TS and energy content and on colostrum yield are presented in Table 4. The farm had a statistically significant effect (*p* < 0.05) on all traits examined.

Fat: Age at calving had a significant effect on colostrum fat content (*p* < 0.05); first, parity cows had higher colostrum fat content than cows in greater parities (*p* < 0.001). Moreover, colostrum from cows that calved in the spring had the greatest fat concentration, while the colostrum from those that calved in the summer and autumn had the lowest (*p* < 0.05). Dry period length had a statistically significant effect on colostrum fat concentration (*p* < 0.05). A dry period ≥ 85 days resulted in a higher fat concentration than a length ≤ 45 days (*p* < 0.05). The time interval between calving and colostrum collection had a significant effect on fat content, as well (*p* < 0.05): the shorter the interval, the higher the concentration. The colostrum yield, previous lactation 305-d milk yield and BCS had no significant effect on colostrum fat content (*p* > 0.05).

Protein: Age at calving had a significant effect on the colostrum protein content (*p* < 0.05); the colostrum from cows with a parity greater than four had the highest protein concentration (*p* < 0.05). Moreover, the colostrum from cows that calved in the autumn and winter had a higher protein concentration than from those that calved in the summer and spring (*p* < 0.05). The time interval between calving and colostrum collection had a significant effect on protein content, as well (*p* < 0.01); as was the case with fat content, the shorter the interval, the higher the concentration. The colostrum yield also had a statistically significant effect on protein content (*p* < 0.001); cows producing < 8.5 kg had higher protein concentrations (*p* < 0.001). Dry period length, previous lactation 305-d milk yield and BCS had no significant effect on colostrum protein content (*p* > 0.05).

Lactose: Age at calving had a significant effect on colostrum lactose content (*p* < 0.05); this time, colostrum from cows with parity greater than four had the lowest lactose concentration (*p* < 0.05). Furthermore, cows that calved in the autumn produced colostrum with the lowest lactose concentrations (*p* < 0.01). The time interval between calving and colostrum collection and colostrum yield had a significant effect on lactose content, as well (*p* < 0.001); the colostrum from cows milked ≥6 h after calving or producing ≥8.5 kg had the highest lactose content (*p* < 0.01). Moreover, colostrum produced from cows with a dry period length of 46–64 days had the highest lactose content and those with ≥85 days had the lowest lactose content, respectively (*p* < 0.05). The previous lactation 305-d milk yield and BCS had no significant effect on colostrum lactose content.

Energy content: Age at calving had a significant effect on colostrum energy content (*p* < 0.05); colostrum from cows with parities of one and greater than four had the highest energy concentrations (*p* < 0.05), while those with parities of two and three had the lowest (*p* < 0.05). Moreover, colostrum from cows that calved in the spring had the highest energy content, and those that calved in the autumn and summer the lowest energy content (*p* < 0.05). Furthermore, cows with a dry period length of ≥65 days had the highest colostrum energy content (*p* < 0.05), and the same was true for cows that were milked ≤2 h after calving (*p* < 0.001). Colostrum yield, previous lactation 305-d milk yield and BCS had no statistically significant effect on its energy content (*p* > 0.05).

Total Solids: Age at calving had a significant effect on colostrum TS content (*p* < 0.05); colostrum from cows with a parity greater than four had the highest TS concentration, and the colostrum from parity two cows had the lowest TS concentration (*p* < 0.01). Cows in parities one and three produced colostrum with intermediate values (*p* < 0.05). Moreover, cows that calved in the autumn had the highest TS concentration, and those that calved in the spring and summer the lowest colostrum TS concentration (*p* < 0.05). Furthermore, colostrum from cows with a time interval between calving and milking ≥6 h had the lowest TS content (*p* < 0.01); this was also the case with cows producing >8.5 kg of colostrum (*p* < 0.001). Dry period length, previous lactation 305-d milk yield and BCS had no statistically significant effect on TS content (*p* > 0.05).

Colostrum yield: Age at calving had a significant effect on colostrum yield (*p* < 0.05); parity one cows had the lowest colostrum yield (*p* < 0.05). Furthermore, cows that calved in the autumn and winter had a lower colostrum yield than those that calved in the spring and summer (*p* < 0.01). Cows with a dry period length of ≤45 days had the lowest colostrum yield (*p* < 0.05). Additionally, cows with a time interval between calving and milking ≥6 h produced significantly more colostrum (*p* < 0.05). Cows with a previous lactation 305-d milk yield ≥ 11,000 kg produced more colostrum than those with milk yield ≤ 7000 kg (*p* < 0.001). Finally, BCS had a significant negative effect on colostrum yield (*p* < 0.05); cows with a BCS > 3.50 produced less colostrum.

## 4. Discussion

Most of the studies have focused on the immunoglobulin content of colostrum with little consideration for other constituents [8,9]. This is reasonable due to the great importance of passive transfer of immunity in calf survival; however, colostrum is also the main source of nutrients for the newborn calf. Colostrum fat is the main energy source for calves, which are born with reduced energy reserves [29]. Depending on the ambient temperature, the latter provides energy for heat production to maintain thermogenesis from a few hours up to several days after birth [30]. Furthermore, colostrum proteins are important for gluconeogenesis during the first 24 h of life [22], which is fundamental for glucose production, the key energy source used by the brain [31].

This was a large-scale study which included more than 1000 cows; it also included a large number of cows per herd, which is in contrast with previous studies where 1239 cows from 21 herds and 827 cows from 67 herds were used, respectively [7,26]. In addition, only *Holstein* cows were included in the present study whereas in other studies *Friesian*, *Ayrshire*, *Swedish Red* crossbreds [7] and *Jersey* crossbreds [7,26] were included as well. In large sample size studies, it is easier to detect a difference between the sample mean and population mean. Moreover, large sample size better represents the population. Furthermore, standard error is directly dependent on sample size.

In the present study, the colostrum yield was very similar to that reported by Kehoe et al. [32] and about 8% lower than that reported by Conneely et al. [9]. Quigley et al. [33] reported a mean colostrum yield of 9.5 ± 3.2 L but the mean collection time after calving was 6.1 ± 3.2 h whereas, in our study, the mean collection time was 3.9 ± 3.2 h. In all cases, the yield variability was high (>30%).

The mean colostrum fat content was similar to that previously reported [7,26]. Overall, the variability of colostrum fat content was 52.3% in the study and ranged from 52% to 62% in previous studies. Interestingly, the colostrum fat content was highly variable within herd. As colostrum fat is the main energy source of newborn calves, this finding indicates that colostrum should be evaluated not only for its IgG content but for its fat content as well.

The mean colostrum protein content was about 30% higher than that reported in previous publications [4,7,26]. This discrepancy is difficult to explain because comparisons among studies are almost impossible due to differences in colostrum definition and yield, the time interval between calving and first milking, cow genetics, regions and rearing systems. The sampling time after calving differed in one study [26], and colostrum yield is not reported in two of the studies [7,26]. Dry cow management is not reported in two of the studies [4,26], while in the Dunn et al. [7] study, management (grazing) was quite different from our study. Mann et al. [34] found that nutritional management during the dry period affects IgG content of colostrum and this may apply for protein content as well. The overall variability of the colostrum protein content was moderate (22.3%) in the present study and within the range reported previously (22% to 26%) [4,7,26]. Within herd variability ranged from 18.2% to 34.0% and was quite lower than that of fat content.

The mean colostrum lactose content was somewhat lower than that reported in previous publications [4,7,26]. The reasons discussed above for colostrum protein content apply here, too. The overall and mean within herd variability (34.0% and 39.7%, respectively) were at the higher ranges (17.0% to 34.0%) of those reported in previous studies.

Furthermore, the mean colostrum TS content in the present study (measured with a Brix refractometer) was 25.8 ± 4.7%, very similar with that reported in other studies (23.8%, 33; 24.3%, 21; 26.3%, 20). Kehoe et al. [4] and Morrill et al. [26] reported mean TS contents (measured by MilkoScan) of 27.6% and 22.2%, respectively. A normal distribution of Brix values is not the rule; this was the case in the present study and in that of Bielmann et al. [20] but not in the study reported by Quigley et al. [33]. The percentage of poor-quality samples (<22%) was 19.2% in the present study, which was in the range previously reported [20,33].

Publications referring to the colostrum energy content are limited. Quigley [35] reported that the mean colostrum metabolizable energy (ME) was 24 MJ/kg of dry matter (5.73 Mcal/kg) and it ranged from 14.2 to 34.8 MJ/kg (3.39 to 8.31 Mcal/kg). Moreover, according to Van Amburgh et al. [30], the colostrum contains approximately 5.67 Mcal of ME/kg of dry matter. Considering the NRC (2001) conversion coefficient (0.86) of metabolizable to net energy and the colostrum dry matter content in this study, the colostrum energy content reported in the different studies is almost identical.

In our study, the herd had a statistically significant effect on all colostrum components and yield. This reflects differences in management practices including cow feeding [7,34] and vaccination [7,36]. Unfortunately, only one herd in our study applied a dry cow vaccination program. Moreover, the nutritional program during the dry period was remarkably similar in all herds in the present study, and typical of almost all dairy herds in Greece. As a consequence, this factor introduced no variation among herds. Cow genetics are another factor that may affect colostrum traits [33], and this was recently confirmed [37]. This may also be the reason why the variance explained by models used in this study were rather low (R^2^s ranged from 0.12–0.29). Unfortunately, R^2^s are not reported in similar studies and no comparisons are possible.

One additional factor that was addressed in the present study was cow BCS at calving, which affected colostrum yield but not composition. Shearer et al. [38] found that an increase in BCS during the dry period was associated with increased colostrum IgG content, but this was not confirmed by Denholm et al. [36]. Our finding must be interpreted with caution in this regard. In the present study, BCS was evaluated only once, at calving; one record of BCS is not representative of the nutrient balance during the dry period [36]. The estimation of BCS change before calving may be a more useful factor in future studies.

The effect of calendar season on colostrum fat and protein content in the present study was similar with that reported by Dunn et al. [7]. Colostrum fat content was in both cases higher in the spring and lower during the summer and autumn, despite the marked difference in management systems in the two studies (year-round housing vs. grazing). Moreover, cows that calved in autumn and winter had the highest colostrum protein content in both studies. A high ambient temperature has been found to negatively affect colostrum fat and protein content in primiparous cows [39]. Heat stress negatively affects milk composition and a similar effect on colostrum cannot be excluded.

In agreement with previous studies [7,26,40] colostrum fat content was highest in primiparous cows and within the range reported. A lower colostrum yield of this age group is usually the reason cited, but no association between colostrum yield and fat content was found in the present study or by Dunn et al. [7]. Moreover, the colostrum protein content increased as parity increased. This is in agreement with some previous studies [7,41] but not others [26,40]. Management differences across studies, different characteristics of colostrum samples (fresh, frozen or refrigerated) [26], different breed composition of the sampled populations and sample size [40] may explain the inconsistency.

In our study, cows that had a dry period longer than 85 days had higher colostrum fat content than cows with a dry period length of less than 45 days. The same effect was observed by Dunn et al. [7] but for dry period lengths >112 and <56 days, respectively. There is no clear reason for this. Colostrum contains a high proportion of long chain fatty acids [23]. Long dry periods are generally associated with elevated BCS and increased mobilization of body energy reserves (that provide long-chain fatty acids) pre-calving. Whether there is a connection between these two facts must be investigated. The positive effect of dry period length on protein content [7] was not confirmed in our study. This may be due to the small number (7%) of cows in our data base that had a dry period length >116 days, but since the number of cows with such a long dry period is not reported in the Dunn et al. [7] study either, no comparisons are possible.

As the time interval between calving and first milking increased in our study, the colostrum fat and protein contents decreased; the inverse relationship (dilution effect) between the lactose and fat colostrum content that was also observed may explain this; however, while the dilution effect was evident for IgG concentration, this was not the case for fat content in a previous study [7].

Contrary to findings of Dunn et al. [7], the colostrum yield negatively affected colostrum protein content in the present study. However, the colostrum yield and its variability, although recorded and analyzed as a factor affecting colostrum composition, are not reported in the Dunn et al. [7] study, and therefore, the comparison cannot be further examined.

An effect of previous lactation milk yield on colostrum protein content was not found in this study, again contrary to the findings of Dunn et al. [7]. The range of previous lactation 305-day milk production in the latter study is not reported either to allow further comparisons between the two studies. Quite possibly, milk production was lower in the grazing herds in the study of Dunn et al. [7] compared to our housed cows’ yield of 9211 ± 1213 kg (Table 1).

The colostrum energy content was affected by herd, calendar season, parity, dry period length and time interval from calving to milking. These are exactly the same factors affecting fat content, as well. This makes sense since fat constitutes the main energy source (≈50%) in colostrum. No other publications referring to colostrum energy content and to factors affecting it were found.

Factors affecting colostrum TS content measured with a Brix refractometer have never been evaluated before. As this is a reliable proxy for colostrum immunoglobulin content [20,21], the comparison with studies evaluating factors affecting the latter reveals notable similarities between the two approaches.

Cows that calved in autumn had a higher content and hence, a better colostrum quality. Higher colostrum IgG content during the same season is reported by several researchers [8,9,42]. On the other hand, Dunn et al. [7] found that best quality colostrum was produced by cows that calved in winter months. In contrast, Pritchett et al. [15] reported that season did not affect colostrum IgG concentration. However, calendar season is a tricky factor; until direct impact of weather conditions is examined, any comparisons are dubious, at least.

Cows with a parity greater than four had the highest colostrum TS content in our study. The increased colostrum IgG content was evident from parity three onwards in several previous studies, both on Holsteins [15,43,44] and other breeds of cows [7,9,26]. This is expected as older cows are exposed to antigens for a longer time, and therefore, they produce and transfer greater antibody quantities to their colostrum [45]. However, the most interesting findings are related to first and second parity cows. It is often proposed that first parity cows, from an immunological perspective, produce low quality colostrum. This was the case in some studies [15,26] but not in others, as colostrum from first parity cows has been found to be of adequate [7,8] or excellent quality [9,44]. In our study, the colostrum TS content of first parity cows did not differ from that of third parity cows (Brix values 25.84 vs. 25.94, percent of samples < the 22% threshold, 17% vs. 18%). Second parity cows had the lowest TS content (Brix value: 24.72%, percent of samples < the 22% threshold: 28%) of all parities, and the differences were statistically significant. No obvious explanation is available for this finding. However, the overall colostrum quality of second parity cows was not actually low; the statistical difference between the first and second parity cows probably resulted from the former’s excellent quality.

Colostrum yield and time interval between calving and first milking had a significantly negative effect on colostrum TS content in this study. Similar negative effects on colostrum IgG content were previously reported [9,17,44] for both factors, as well. In the Dunn et al. [7] study, there was no significant effect of colostrum yield, but longer time intervals between calving and first milking negatively affected the colostrum IgG content. Again, these findings indicate that as yield and time from calving increases, the colostrum dilution effect is taking place. Different authors, based on their findings, suggest different time points that colostrum IgG content becomes low enough to be considered inadequate. Moore et al. [44], Quigley et al. [33] and Conneely et al. [9] reported that this happened 6, 8 and 9–12 h after calving, respectively; Dunn et al. [7] found a significant negative effect beyond 12 h after calving. In the present study, colostrum TS content decreased significantly when the time interval from calving to first milking exceeded 6 h. Therefore, the colostrum should be collected as soon as possible after calving.

We found no significant effect of dry period length on colostrum TS content. Continuous milking (no dry period) is known to result in lower colostrum immunoglobulin content [46,47]). A dry period length of <8 weeks was previously found to negatively affect colostrum IgG content [7] but actual dry period length was not reported. Other studies reported no differences between 60-d and 30-d dry periods [46,47].

Overall, the same factors found to affect the colostrum immunoglobulin content appear to affect the colostrum TS content measured with a Brix refractometer, enhancing the usefulness of the latter as an on-field management tool.

## 5. Conclusions

From a nutritional standpoint, the fact that the colostrum fat content was highly variable corroborates the need for its evaluation before feeding to newborn calves. The protein content was not as variable, but its significant association with passive transfer of immunity make its evaluation very useful, as well. Several factors affecting colostrum composition were identified, namely parity, season, colostrum yield and the time interval between calving and colostrum collection, offering prospects in colostrum management in order to improve calf health in dairy herds.

## Figures and Tables

**Table 1:** Factors evaluated for their effect on colostrum composition.

Factors	Observations	Mean	SD	Min	Max
Dry period length (days)	687	67	27	1	213
Milk yield of previous 305-d lactation (kg)	640	9211	1913	3136	16,273
Age at calving (months)	1074	46.5	22.5	19.1	151.3
Body Condition Score at calving	1074	2.93	0.50	1.75	4.75
Interval calving colostrum collection	1074	232	195	10	960

**Table 2:** Descriptive statistics of colostrum traits.

Trait	Observations	Mean	SD	CV (%)	Median	Lower Quartile	Upper Quartile
Fat (%)	1074	6.37	3.33	52	5.96	2.54	10.92
Protein (%)	1074	17.83	3.97	22	17.88	12.67	22.70
Lactose (%)	1074	2.15	0.73	34	2.16	1.23	3.06
Total solids (%Brix value)	1074	25.80	4.68	18	25.90	19.76	31.55
Energy (Mcal/kg)	1074	1.35	0.29	21	1.33	0.99	1.74
Colostrum yield (kg)	1074	6.20	3.80	61	5.00	3.50	8.00

**Table 3:** Among and within farm variability of colostrum yield and composition.

Farm	Fat	Protein	Lactose	Total Solids	Energy	Yield
	(%)	(%)	(%)	(%Brix Value)	(Mcal/kg)	(kg)
	Mean (SD)					
1	3.00	16.98	1.47	22.80	1.04	5.00
	(1.46)	(3.12)	(1.20)	(5.40)	(0.26)	(2.97)
2	5.33	16.72	2.39	24.55	1.23	7.70
	(3.33)	(3.83)	(0.67)	(4.29)	(0.27)	(3.89)
3	6.61	17.67	2.02	25.81	1.36	5.30
	(3.53)	(3.70)	(0.68)	(4.20)	(0.29)	(2.96)
4	7.50	18.04	2.09	26.50	1.44	6.40
	(3.35)	(3.94)	(0.71)	(4.84)	(0.29)	(4.17)
5	7.69	17.54	2.01	25.45	1.43	6.20
	(3.41)	(3.43)	(0.66)	(4.11)	(0.28)	(2.61)
6	5.33	16.12	2.24	23.27	1.20	12.60
	(3.33)	(4.82)	(0.90)	(5.23)	(0.26)	(4.14)
7	6.06	18.14	2.19	26.03	1.34	5.60
	(3.09)	(3.90)	(0.74)	(4.64)	(0.29)	(3.64)
8	5.52	18.46	2.06	26.85	1.31	5.70
	(2.61)	(4.42)	(0.75)	(5.06)	(0.27)	(4.03)
9	5.99	19.42	2.20	26.59	1.40	5.40
	(3.23)	(3.85)	(0.73)	(4.87)	(0.31)	(1.36)
10	5.86	19.84	2.24	23.36	1.27	5.30
	(2.60)	(5.72)	(0.98)	(6.41)	(0.31)	(3.90)

**Table 4:** Factors affecting colostrum quality and yield.

Factor	Size of Effect					
	Fat (%)	Protein (%)	Lactose (%)	Energy (Mcal/kg)	Total Solids (%Brix)	Yield (kg)
Parity	**	*	*	*	*	*
1	7.77 ^b^	17.15 ^a^	2.24 ^b^	1.36 ^b^	25.38 ^b^	4.95 ^a^
2	5.50 ^a^	16.93 ^a^	2.36 ^b^	1.35 ^a^	24.40 ^a^	6.75 ^b^
3	5.32 ^a^	17.98 ^a^	2.20 ^b^	1.32 ^a^	25.81 ^b^	7.69 ^b^
4+	5.92 ^a^	18.96 ^b^	1.97 ^a^	1.36 ^b^	26.77 ^c^	6.99 ^b^
Season	*	**	**	*	**	***
Winter	6.11 ^a,b^	18.01 ^b^	2.30 ^b^	1.35 ^a,b^	25.62 ^a,b^	6.01 ^a^
Spring	6.58 ^b^	17.64 ^a^	2.24 ^b^	1.37 ^b^	25.50 ^a^	7.52 ^b^
Summer	6.06 ^a^	17.33 ^a^	2.19 ^b^	1.32 ^a^	25.01 ^a^	6.99 ^b^
Autumn	5.77 ^a^	18.03 ^b^	2.04 ^a^	1.34 ^a^	26.23 ^b^	5.77 ^a^
Milk yield of previous 305-day lactation (kg)	NS	NS	NS	NS	NS	***
≤7000	5.96	17.64	2.05	1.32	24.97	5.61 ^a^
7000–9000	5.35	17.69	2.11	1.37	25.11	6.42 ^a,b^
9000–11,000	5.10	18.26	2.00	1.37	25.81	7.39 ^b,c^
≥11,000	5.61	18.01	2.14	1.39	25.61	8.53 ^c^
Dry period length (days)	*	NS	*	*	NS	*
≤45	5.07 ^a^	18.33	2.02 ^a,b^	1.34 ^a^	25.68	6.10 ^a^
46–64	5.12 ^a^	17.41	2.22 ^b^	1.35 ^a^	24.66	6.66 ^a,b^
65–84	5.45 ^a,b^	17.70	2.06 ^a,b^	1.39 ^a,b^	25.07	7.45 ^a,b^
≥85	6.37 ^b^	18.14	1.99 ^a^	1.38 ^b^	26.09	7.74 ^b^
Colostrum Yield (kg)	NS	***	***	NS	**	
≤4.0	5.83	18.46 ^b^	2.03 ^a^	1.37	26.33 ^b^	
4.1–8.5	6.25	18.06 ^b^	2.19 ^b^	1.33	25.94 ^b^	
>8.5	6.30	16.75 ^a^	2.35 ^c^	1.34	24.51 ^a^	
Time interval between calving and colostrum collection (h)	*	**	***	*	***	*
≤2	6.55 ^b^	18.24 ^b^	2.07 ^a^	1.38 ^b^	26.18 ^b^	6.14 ^a,b^
2–6	6.09 ^a,b^	17.91 ^b^	2.17 ^a^	1.37 ^b^	25.83 ^b^	6.26 ^a^
≥6	5.74 ^a^	17.11 ^a^	2.34 ^b^	1.34 ^a^	24.76 ^a^	7.39 ^b^
Age at calving	−0.19 *	0.24 *	−0.05 *	−0.02 *	0.20 *	0.47 *
Body Condition Score	0.93 ^NS^	−0.25 ^NS^	0.04 ^NS^	−0.01 ^NS^	−0.17 ^NS^	0.43 *

For Season, Parity, Milk yield of previous 305-day lactation, Dry period length, Colostrum Yield and Time interval between calving and colostrum collection, values shown are marginal means whereas for Age at calving and Body Condition Score are regression slopes. * *p* ≤ 0.05, ** *p* ≤ 0.01, *** *p* ≤ 0.001, effects of factors on colostrum traits. ^a–c^ Means in the same column having different superscripts differ significantly. NS means not statistically significant.

## Data Availability

Additional data are available on request from the corresponding author.
